# Suppression of thyrotropin secretion during roxadustat treatment for renal anemia in a patient undergoing hemodialysis

**DOI:** 10.1186/s12882-021-02304-2

**Published:** 2021-03-20

**Authors:** Mitsuru Ichii, Katsuhito Mori, Daichi Miyaoka, Mika Sonoda, Yoshihiro Tsujimoto, Shinya Nakatani, Tetsuo Shoji, Masanori Emoto

**Affiliations:** 1 Division of Internal Medicine, Inoue Hospital, Suita, Japan; 2 Department of Nephrology, Osaka City University Graduate School of Medicine, 1-4-3, Asahi-machi, Abeno-ku, 545-8585 Osaka, Japan; 3 Department of Metabolism, Endocrinology and Molecular Medicine, Osaka City University Graduate School of Medicine, Osaka, Japan; 4 Department of Vascular Medicine, Osaka City University Graduate School of Medicine, Osaka, Japan

**Keywords:** Renal anemia, Roxadustat, Thyroid stimulating hormone (TSH), Thyroid hormone receptor, Case report

## Abstract

**Background:**

Inhibition of hypoxia-inducible factor prolyl hydroxylase (HIF-PH) is a novel choice for the treatment of renal anemia, and an oral HIF-PH inhibitor roxadustat was approved for renal anemia. Roxadustat has high affinity to thyroid hormone receptor beta, which may affect thyroid hormone homeostasis.

**Case presentation:**

We present here a patient undergoing hemodialysis with primary hypothyroidism receiving levothyroxine replacement, who showed decreased free thyroxine (FT4) and thyroid stimulating hormone (TSH) after starting roxadustat. Pituitary stimulation test revealed selective suppression of TSH secretion. Recovery of TSH and FT4 levels after stopping roxadustat suggested the suppression of TSH was reversible.

**Conclusions:**

Physicians should pay special attention to thyroid hormone abnormalities in treatment with roxadustat.

## Background

Renal anemia is an important complication which could affect quality of life of patients with chronic kidney disease (CKD) including those undergoing hemodialysis [1]. Anemia in CKD results from alterations of iron metabolism and impaired erythropoietin production by the kidneys [2, 3]. The current standard treatment for anemia in patients on dialysis includes use of intravenous iron, use of erythropoiesis stimulating agents (ESA), and combination of them [2, 3]. Hypoxia-inducible factor (HIF) is involved in the impaired erythropoietin production in CKD [4] and rapidly degraded by HIF prolyl hydroxylase (HIF-PH). Thus, stabilization of HIF by HIF-PH inhibition is a novel strategy to renal anemia. Roxadustat, an oral inhibitor of HIF-PH, was shown be non-inferior to darbepoetin alfa [5]. In addition, its dose-effectiveness appears independent of serum C-reactive protein (CRP) levels, which may be advantage over ESA, because ESA-resistance occurs in patients with high CRP levels [6].

Roxadustat has structure similar to triiodothyronine (T3) and also binds to thyroid hormone receptor β (THRβ) at a higher affinity than T3 [7]. Since THRβ in the pituitary and hypothalamus plays an important role in the feed-back regulation of thyroid hormone [8], roxadustat may affect the homeostasis of thyroid hormone.

Here, we describe a hemodialysis patient who showed suppression of thyroid stimulating hormone (TSH, or thyrotropin) and decreased free thyroxine (FT4) levels following roxadustat treatment for renal anemia.

## Case presentation

A 77-year-old man with kidney failure due to undetermined etiology had been treated with hemodialysis for 7 years. He had history of surgery for esophageal cancer and gastric cancer before starting dialysis, and also hepatocellular carcinoma after starting dialysis. He was diagnosed at the age of 74 with subclinical primary hypothyroidism based on high level of TSH (9.5 µIU/mL) with normal level of FT4 (1.0 ng/dL). Hashimoto’s thyroiditis was suspected based on clinical features including diffuse swelling of thyroid gland as well as hypoechoic and heterogenous pattern on thyroid ultrasonographic image, but autoantibodies such as anti-thyroid peroxidase and anti-thyroglobulin antibodies were negative. Since he complained of general fatigue and his laboratory test showed subclinical hypothyroidism, replacement therapy was started with 37.5 µg per day of levothyroxine. In addition to levothyroxine, he was taking 5 mg per day of cilnidipine and 40 mg per day of telmisartan for hypertension, 750 mg per day of lanthanum carbonate for hyperphosphatemia, 1 mg per day of evocalcet for secondary hyperparathyroidism, ‎0.5 mg per day of entecavir hydrate for hepatitis B, 30 mL per day of lactulose syrup 60 % for hyperammonemia, and 3 tables of a butyric acid-producing bacillus preparation for constipation.

**Fig. 1:**
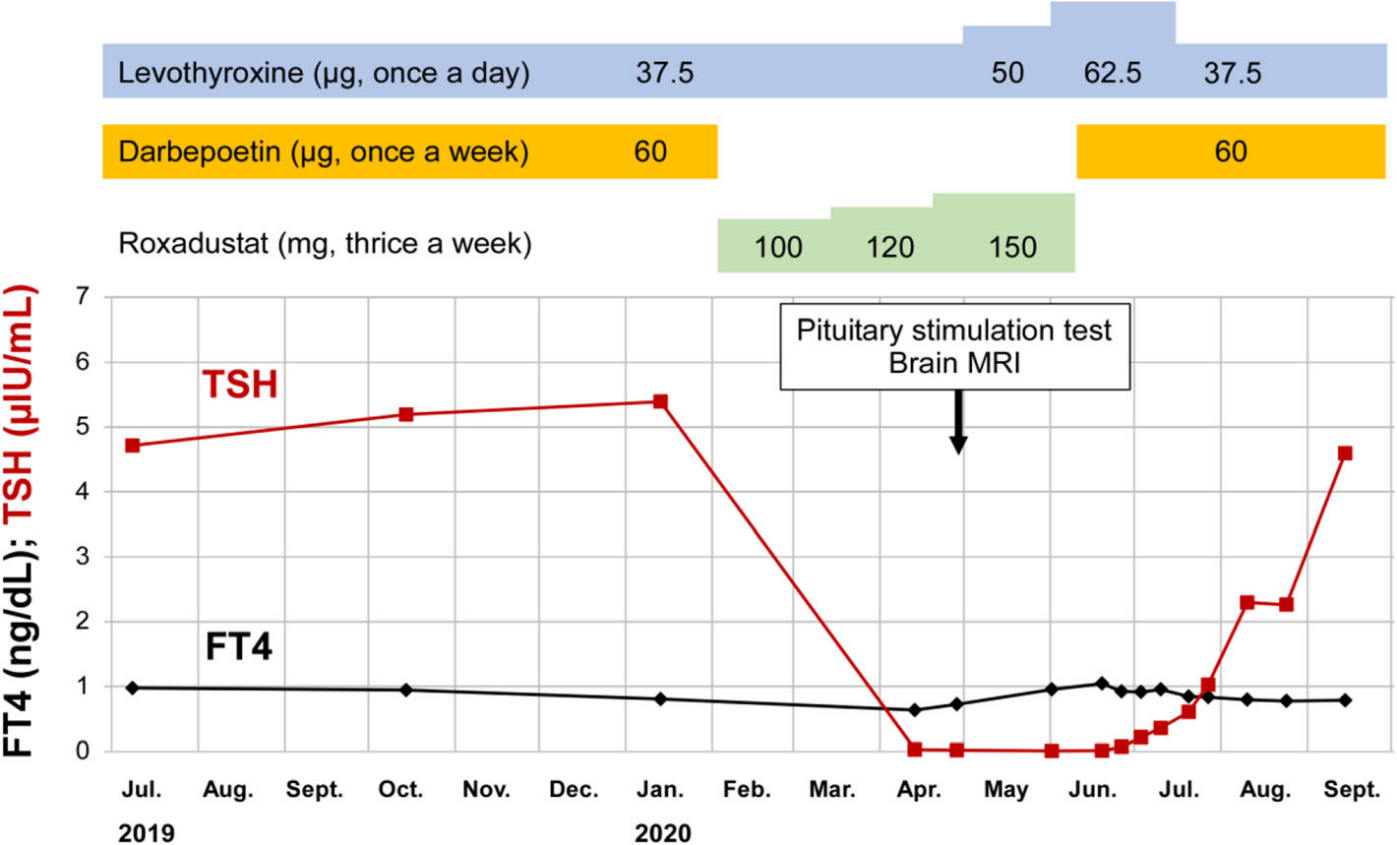
Changes in TSH and FT4 levels in this case. Roxadustat was administered from February 2 to June 12, 2020. Reference ranges of TSH and FT4 were between 0.541 and 4.261 µIU/mL and between 0.76 and 1.65 ng/dL, respectively. Abbreviations: TSH, thyroid stimulating hormone; FT4, free thyroxine; MRI, magnetic resonance imaging

Because his hemoglobin level did not improve despite treatment with 60 µg of darbepoetin α once weekly and no sign of iron deficiency, treatment for anemia was switched to 100 mg of roxadustat thrice weekly. After starting roxadustat, his hemoglobin level remained unchanged and he complained no specific symptom. However, decreased levels of both TSH and FT4 were noticed, as shown in Fig. 1, which was not attributable to known primary hypothyroidism.

Then, anterior pituitary function test was performed by combined intravenous administration of four hypothalamic releasing hormones. As shown in Table 1, the results revealed the selective and severe suppression of TSH response. Since the diagnose of central hypothyroidism was made in addition to the existing primary hypothyroidism, the dose of levothyroxine was increased from 37.5 to 62.5 µg per day, and his FT4 was slightly increased. Magnetic resonance imaging did not show abnormal findings in the hypothalamus-pituitary region.

**Table 1:** Pituitary stimulation tests

Time (minutes)	TSH (µIU/mL)	LH (µIU/mL)	ACTH (pg/mL)	PRL (ng/mL)	COR (µg/dL)	GH (ng/mL)
0	0.017	87.7	44.7	17.4	6.1	2.2
15	0.044	112.4	92.9	34.0	9.0	45.0
30	0.054	123.2	93.6	34.6	10.0	61.3
60	0.054	138.0	85.8	42.6	9.8	35.5
90	0.062	145.9	70.4	39.7	9.0	—
120	0.047	147.0	62.7	36.7	7.6	—

The table gives the responses of the six anterior pituitary hormones to intravenous injection of the four hypothalamic releasing hormones (TRH, LH-RH, CRH, and GH-RP2). Note that the TSH response was selectively suppressed.

Abbreviations: *TSH* thyroid stimulating hormone; *LH* luteinizing hormone; *ACTH* adrenocorticotropic hormone; *PRL* prolactin; *COR* cortisol; *GH* growth hormone; *TRH* thyrotropin releasing hormone; *LH-RH* luteinizing hormone releasing hormone; *CRH* corticotropin releasing hormone; *GH-RP2* growth hormone releasing protein 2

In search of possible signs of thyroid hormone deficiency, we reviewed routine laboratory measurements, vital signs, and body mass index. The results in Table 2 revealed remarkable reductions in serum total cholesterol, high-density lipoprotein cholesterol, and non-high-density lipoprotein cholesterol levels. Monthly-averaged blood pressure and pulse rate levels measured at the start of hemodialysis session were slightly increased following roxadustat treatment. Serum creatinine level showed a reduction, whereas no appreciable changes were found in liver function tests or body mass index calculated from body weight at the end of hemodialysis session. These changes in laboratory tests and vital signs did not support thyroid hormone deficiency.

**Table 2:** Routine laboratory tests, vital signs, and body mass index before, during, and after roxadustat treatment

	2019								2020								
	May	Jun.	Jul.	Aug.	Sept.	Oct.	Nov.	Dec.	Jan.	Feb.	Mar.	Apr.	May	Jun.	Jul.	Aug.	Sept.
Treatment for anemia	DARBE	DARBE	DARBE	DARBE	DARBE	DARBE	DARBE	DARBE	DARBE	ROXAD	ROXAD	ROXAD	ROXAD	DARBE	DARBE	DARBE	DARBE
Hemoglobin (g/dL)	9.4	9.5	9.8	10.6	9.5	8.3	9.3	9.2	9.0	8.5	8.9	9.2	8.8	7.9	8.2	7.2	7.5
TC (mg/dL)	126	124	115	113	120	110	123	129	127	76	67	64	52	67	122	111	126
Non-HDL-C (mg/dL)	88	—	85	—	87	—	91	—	92	—	49	47	33	43	85	80	92
HDL-C (mg/dL)	30	—	30	—	33	—	32	—	36	—	18	17	19	24	37	31	34
AST (IU/L)	18	17	16	18	17	17	22	19	7	18	19	20	17	19	18	17	17
ALT (IU/L)	8	8	8	8	8	8	9	8	8	9	8	10	9	9	7	7	7
Serum albumin (g/dL)	3.6	3.5	3.3	3.4	3.4	3.3	3.5	3.5	3.4	3.0	3.1	3.4	3.4	3.2	3.5	3.2	3.4
BUN (mg/dL)	36.3	54.3	52.2	58.0	47.8	61.2	55.5	60.5	48.6	57.4	48.3	51.6	50.9	40.8	39.4	43.8	54.2
Creatinine (mg/dL)	8.9	8.9	9.7	9.8	9.6	9.7	9.6	9.2	9.5	8.2	8.1	7.6	7.2	6.5	7.0	8.0	8.9
CRP (mg/dL)	0.27	0.68	0.22	0.13	0.37	0.38	0.28	0.19	0.24	0.43	0.30	0.25	0.23	0.28	0.21	0.35	0.15
Ferritin (ng/ml)	—	120.4	—	—	420.3	—	—	339.7	—	361.8	324.0	219.3	193.8	215.6	238.2	178.7	173.8
TSAT (%)	27.5	24.0	21.8	30.2	30.4	45.5	32.3	44.5	50.9	56.2	82.4	41.8	57.1	39.3	40.8	26.8	28.3
BMI (kg/m^2^)	22.1	22.0	21.9	21.9	22.1	22.1	22.2	22.4	22.4	22.4	22.2	21.9	21.9	21.8	21.6	21.8	21.5
Pulse rate (ppm)	64.1	69.6	65.5	67.4	69.5	65.4	68.5	67.1	67.4	71.8	71.7	73.4	69.9	68.6	67.5	65.6	63.8
SBP (mmHg)	146.3	153.3	149.6	139.7	140.4	138.7	147.2	141.2	144.5	150.3	162.7	160.0	153.8	151.4	154.0	151.2	148.0
DBP (mmHg)	70.0	76.2	72.3	68.5	69.5	69.3	71.2	66.6	69.0	74.4	78.4	73.1	70.2	71.4	71.6	71.5	68.2

Treatment for renal anemia was switched from darbepoetin alfa to roxadustat. The treatment with roxadustat was done from February 2 to June 12, 2020. Measurements were done in the last week of each month. Note the reductions of TC, Non-HDL-C, and HDL-C levels during roxadustat treatment.

Abbreviations: *DARBE* darbepoetin alfa; *ROXAD* roxadustat; *TC* total cholesterol; *Non-HDL-C* non-high-density lipoprotein cholesterol; *HDL-C* high-density lipoprotein cholesterol; *AST* aspartate aminotransferase; *ALT*alanine aminotransferase; *BUN* blood urea nitrogen; *CRP* C-reactive protein; *TSAT* transferrin saturation; *BMI* body mass index; *SBP* systolic blood pressure; *DBP* diastolic blood pressure

Because we suspected the possible influences of roxadustat, it was stopped, darbepoetin α was restarted, and the dose of levothyroxine was re-adjusted to 37.5 µg per day. Then, his TSH was increased from 0.006 to 4.592 µIU/mL. His cholesterol, blood pressure, and pulse rate returned to the levels seen before roxadustat was started.

## Discussion and conclusion

We report here a patient undergoing hemodialysis who showed decreased levels of TSH and FT4 levels after starting roxadustat treatment for renal anemia, in whom selective suppression of TSH from the anterior pituitary was demonstrated. The recovery of TSH after withdrawal of roxadustat suggests that the suppression of TSH is reversible.

Thyroid hormone is tightly controlled by the feed-back regulation in the hypothalamus-pituitary-thyroid axis, and THRs play essential roles in the actions of thyroid hormone. THR and retinoid X receptor (RXR) form a heterodimer at the thyroid hormone responsive element of genes, and binding of T3 to THR promotes coactivator binding and gene transcription [9]. THR has isoforms with different tissue distributions accounting for tissue-specific action of thyroid hormone. THRβ plays an important role in hypothalamus and pituitary gland. According to Yao et al., roxadustat has a molecular structure similar to T3, and it binds to THRβ at a higher affinity than T3 [7]. In addition, THRβ has a predominant role in the down-regulation of thyrotropin releasing hormone (TRH) secretion from hypothalamus and TSH secretion from the anterior pituitary gland than THRα[8]. Therefore, it is conceivable that roxadustat acts as an agonist of THR β, suppresses TSH secretion, and results in decreased serum FT4 level, as noticed in this case.

Perturbation of TSH by medication is known for other drugs. Bexarotene, used for treatment of cutaneous T-cell lymphoma, is known to cause central hypothyroidism [10]. Bexarotene has the structure of an RXR selective ligand. Binding of bexarotene with RXR promotes heterodimer complex formation with THR, inhibits secretion of TSH, and central hypothyroidism in more than 90 % of patients [11]. Nineteen of 27 patients noted that fatigue and cold intolerance, which were improved by levothyroxine [11]. Central hypothyroidism caused by bexarotene can be reversed by stopping the drug [10]. In contrast, nivolumab [12] and ipilimumab [13] can cause irreversible central hypothyroidism by inducing hypophysitis by an autoimmune mechanism.

The abnormal thyroid function tests observed in our patient were similar to the bexarotene-induced central hypothyroidism, but pathophysiologic implications may be different. Namely, the thyroid function tests in our patient do not necessarily indicate central hypothyroidism, because roxadustat could act as an agonist of THRβ not only in the hypothalamus and the pituitary gland, but also in peripheral tissues. Central hypothyroidism due to bexarotene was accompanied by hyperlipidemia [11] similar to elevated cholesterol and triglyceride levels often seen in primary hypothyroidism [14]. In contrast, the current case showed a reduction of serum cholesterol level after starting roxadustat, suggesting the excess rather than insufficiency of thyroid hormone function. In addition, the observed increase in blood pressure and pulse rate also supports this notion. Although reduced serum creatinine level is known for hyperthyroidism [15], the decreased serum creatinine in this case with kidney failure undergoing hemodialysis may be attributable not to increased kidney function but to alterations in skeletal muscles, which can be caused by the agonistic action to THR of roxadustat or by changes in thyroid hormone levels. Thus, we speculate the changes in thyroid hormone tests during roxadustat treatment indicate T3-mimetic action of roxadustat and excessive thyroid hormone functions in various tissues.

There are a few points which remain to be clarified. First, although decreased FT4 was treated by increasing the dose of levothyroxine in this case based on the diagnosis of central hypothyroidism in addition to the existing primary hypothyroidism, we are not sure that the diagonosis was correct. If roxadustat exerted its thyromimetic actions in this case, thyroid hormone replacement should have been reduced even when the level of FT4 was very low due to “pseudo” central hypothyroidism. Second, the cholesterol-lowering effect of roxadustat was known in the previous clinical trials in patients with anemia undergoing hemodialysis [6, 16], but its effects on thyroid hormone were not reported. Therefore, the possible link between thyroid hormone abnormalities and reduction in cholesterol following roxadustat may be worth exploring, but it is not straight-forward. Other possible explanations include reduced cholesterol synthesis due to increased degradation of 3-hydroxy-3-methylglutaryl-coenzyme A reductase stimulated by a mechanism mediated by HIF [17]. And third, since no adverse effect on thyroid hormone was reported in many patients in the clinical trials, the reason why this case was affected is unclear. The multiple comorbidities (including primary hypothyroidism, hepatitis, and cancer history) of this patient could be a factor which determined the susceptibility. Such patients with multiple comorbidities are likely to be excluded from clinical trials.

In conclusion, this is the first report of a case with renal anemia showing that treatment with roxadustat could interfere with the homeostasis of the hypothalamus-pituitary-thyroid axis. Further information is needed for safe treatment of renal anemia with this novel agent.

## Data Availability

MI examined the patient. MI has full access to all data in this case report.

## Abbreviations

CKD: Chronic kidney disease
ESA: Erythropoiesis stimulating agents
HIF: Hypoxia-inducible factor
HIF-PH: HIF prolyl hydroxylase
CRP: C-reactive protein
T3: Triiodothyronine
THR: Thyroid hormone receptor
TSH: Thyroid stimulating hormone
FT4: Free thyroxine
RXR: Retinoid X receptor
TRH: Thyrotropin releasing hormone
MRI: Magnetic resonance imaging
